# BU-32: a novel proteasome inhibitor for breast cancer

**DOI:** 10.1186/bcr2411

**Published:** 2009-10-12

**Authors:** Joseph K Agyin, Bindu Santhamma, Hareesh B Nair, Sudipa S Roy, Rajeshwar R Tekmal

**Affiliations:** 1 Department of Biochemistry, University of Texas Health Science Center at San Antonio, 7703 Floyd Curl Drive, San Antonio, TX 78229, USA; 2 Department of Obstetrics & Gynecology, University of Texas Health Science Center at San Antonio, 7703 Floyd Curl Drive, San Antonio, TX 78229, USA

## Abstract

**Introduction:**

Proteasome inhibition provides an attractive approach to cancer therapy and may have application in the treatment of breast cancer. However, results of recent clinical trials to evaluate the effect of the proteasome inhibitor Bortezomib (Velcade^®^, also called PS-341) in metastatic breast cancer patients have shown limited activity when used as a single agent. This underscores the need to find new and more efficacious proteasome inhibitors. In this study, we evaluate the efficacy of the novel proteasome inhibitor BU-32 (NSC D750499-S) using *in vitro *and *in vivo *breast cancer models.

**Methods:**

We have recently synthesized a novel proteasome inhibitor (BU-32) and tested its growth inhibitory effects in different breast cancer cells including MCF-7, MDA-MB-231, and SKBR3 by *in vitro *cytotoxicity and proteasomal inhibition assays. The apoptotic potential of BU32 was tested using flow cytometry and analyzing cell cycle regulatory proteins. *In vivo *tumor xenograft studies for solid tumor as well as tumor metastasis were conducted using MDA-MB-231-GFP cells.

**Results:**

We report for the first time that BU-32 exhibits strong cytotoxicity in a panel of cell lines: MDA-MB-231 (IC_50 _= 5.8 nM), SKBR3 (IC_50 _= 5.7 nM) and MCF-7 cells (IC_50 _= 5.8 nM). It downregulates a wide array of angiogenic marker genes and upregulates apoptotic markers, including Bid and Bax. Incubation of MDA-MB-231 cells with BU-32 results in the accumulation of cell cycle inhibitor proteins p21 and p27 and stabilization of the tumor suppressor protein p53. Studies in *in vivo *solid tumor and metastasis models show significant effect with a 0.06 mg/kg dose of BU-32 and marked reduction in tumor burden in the skeleton.

**Conclusions:**

We have shown that BU-32 is effective in cultured breast cancer cells and in breast cancer xenografts. The results suggest its potential benefit in breast cancer treatment.

## Introduction

The proteasome is a multi-catalytic, multi-subunit protease complex that is responsible for the ubiquitin-dependent turnover of cellular proteins [1-3]. The proteolytic component of this system, the 26S proteasome, consists of two 19S regulatory particles, involved in substrate recognition and unfolding, and a core particle, the 20S proteasome [4]. The proteolytic activity of the proteasome measured against fluorogenic substrates illustrates three distinct cleavage preferences, termed chymotryptic-like activities, tryptic-like activities, and caspase-like activities [5,6]. Catalytic activity of each proteasome active site is associated with the N-terminal threonine residue, which acts as a nucleophile in hydrolysis [3,7,8]. Since proteasomes play a central role in the cytoplasmic turnover of the vast majority of proteins, the manipulation of proteasomal activity is a key goal in controlling the stability of regulatory proteins [3,9]. Inhibition of the proteasome results in abnormal accumulation of many intracellular proteins, thereby disrupting cellular homeostasis [10], and results in the induction of tumor cell apoptosis [11,12].

The most studied and best characterized proteasome inhibitor is Bortezomib (PS-341, Velcade^®^; Millenium Pharmaceuticals Inc., Cambridge, MA, USA and Johnson Pharmaceutical Research and Development, LLC, Raritan, NJ, USA), a dipeptide boronic acid that works by reversibly inhibiting the effects of the proteasome and inducing apoptosis in several tumor cell lines and animal models [13-15]. Bortezomib has a wide range of molecular effects, including stabilization of cell cycle regulatory proteins, inhibition of NF-κB activation, induction of apoptosis, and override of Bcl-2 resistance and angiogenesis [14,16]. The mechanism of action of Bortezomib has been shown to involve the inhibition of the β_5_-subunit (chymotrypsin-like activity) and the β_1_-subunit (caspase-like activity), with the β_5_-subunit as the predominant target [17].

Bortezomib has been approved by the US Food and Drug Administration for the treatment of chemorefractory multiple myeloma patients [18-20] and for some forms of non-Hodgkin's lymphoma [21,22], and the inhibitor is in further clinical development in multiple tumor types, including breast cancer [23-25]. Despite its clinical success, dose-limiting toxicities including grade 4 arthralgia, diarrhea, vomiting, grade 3 thrombocytopenia, anemia, febrile neutropenia, gastrointestinal toxicity, pain, fatigue, neuropathy, and electrolyte disturbances [26-28] have restricted Bortezomib to a twice-weekly day 1/day 4 dosing schedule to allow complete recovery of proteasome activity between doses [26-29].

These observations suggest that the search for additional proteasome inhibitors is warranted. We have recently designed and synthesized a new proteasome inhibitor, BU-32, a bis-dipeptidyl boronic acid analog of Bortezomib containing an additional dipeptide boronic acid moiety on the pyrazine ring, intended to potentially achieve stronger binding affinity and increased potency . Bivalent proteasome inhibitors, either hetero-bivalent or homo-bivalent, have been reported to increase inhibition potency by as much as two orders of magnitude compared with the monovalent analogs, although in these compounds the active moieties are typically separated by a linker of 18 to 22 carbon atoms long [30-32]

In the present study, we describe the *in vitro *and *in vivo *characterization of BU-32 in breast cancer cell lines and xenograft and metastatic models. In order to test the activity of BU-32, irrespective of estrogen receptor status, we used a panel of breast cancer cell lines: MCF-7 (estrogen receptor-positive, progesterone receptor-positive), MDA-MB-231 (estrogen receptor-negative, progesterone receptor-negative, HER2-negative) and SKBR3 (HER-positive). We show that BU-32 is a potent and selective inhibitor of the chymotrypsin-like activity of the proteasome *in vitro*. In addition, we show that BU-32 modulates cell-cycle-dependent kinase inhibitors, upregulates p53 and proapoptotic factors Bax and Bid, downregulates NF-κB expression at the protein level, induces apoptosis, and inhibits various angiogenic factors in a panel of breast cancer cell lines. In addition, we show that BU-32 has *in vivo *anti-tumor activity and inhibits breast cancer-initiated bone metastasis in experimental animals.

## Materials and methods

### Proteasome inhibitor BU-32

BU-32 (pyrazyl-2,5-*bis*-CONH(CHPhe)CONH(CHisobutyl)B(OH)_2_) was synthesized in our laboratory using standard synthetic procedures and was characterized by ^1^H and ^13^C nuclear magnetic resonance and mass spectrometry. The detailed synthetic procedure will be reported elsewhere. BU-32 was dissolved in dimethylsulfoxide and stored at -20°C until use. BU-32 was diluted in culture media immediately before use. Both BU-32 and control media contained < 0.1% dimethylsulfoxide.

### Cell culture

MCF-7 and MDA-MB-231 human breast cancer cells were maintained in DMEM supplemented with 10% FBS. SKBR3 human breast cancer cell line obtained from the American Type Culture Collection (Manassas, VA, USA) was maintained in McCoys5A, 10% FBS.

### Animals

Two-week-old to 4-week old Balb/c female nude mice (obtained from Harlan Sprague Dawley, Inc., Indianapolis, IN, USA) were used in the *in vivo *animal experiments. All animal protocols were approved and monitored by the Institutional Animal Care and Use Committee. The animals were housed under specific pathogen-free conditions.

### Cell viability

Cell viability was determined by quantification of 3-(4,5-dimethylthiazol-2-yl)-2,5-diphenyltetrazolium bromide (MTT) reduction by mitochondrial dehydrogenases. In brief, cells (4 × 10^4 ^cells per well) were incubated with 4 to 18 nM Bortezomib and BU-32 for 48 hours. Thereafter, MTT in a final concentration of 1.2 mmol/l was added and further incubated for 3 hours at 37°C. The formazan dye solubilized in 20% SDS in 50% dimethyl formamide was added and the plate was incubated at room temperature for 1 hour. The absorption was measured at 550 nm versus 690 nm in a microplate reader (Molecular Devices, Corning, New York, USA).

### 20S proteasome assay

Proteasome chymotrypsin-like activities, caspase-like activities, and trypsin-like activities were determined using the Proteasome-Glo™ Assay System (Promega, Madison, Wisconson, USA) according to the manufacturer's protocol. Proteasome-Glo™ buffer was mixed with luciferin detection reagent, and then the substrate was added to the mixture and incubated at room temperature for 1 hour. An equal volume of proteasome-Glo™ reagent was added to the samples and further incubated for 15 minutes. The luminogenic substrate containing the Suc-LLVY sequence is recognized by the proteasome. Following proteasome cleavage, the substrate for luciferase (aminoluciferin) was released, allowing the luciferase reaction to proceed and produce light. The luminescence was measured with a luminometer (Thermo Fisher Scientific, Waltham, MA, USA).

### Western blotting

Cell lines were harvested into a lysis buffer containing a cocktail of protease inhibitors (Sigma, St Louis, MO, USA). The protein content was assessed by the Bradford assay. Equal protein concentrations were prepared for loading with Laemmli sample buffer and were run on SDS-PAGE. Separated proteins were then transferred to nitrocellulose membrane, and the protein-bound membranes were incubated for 2 hours at room temperature with Tris-buffered saline containing 0.05% Triton X-100 and 5% nonfat dry milk to block nonspecific antibody binding. The membranes were then incubated with the respective primary antibodies in Tris-buffered saline milk overnight at 4°C, and specific binding was visualized using species-specific IgG followed by enhanced chemiluminescent detection (ECL kit; Amersham Bioscience, Pittsburg, PA, USA) and exposure to enhanced chemiluminescent X-ray film. Antibodies against β-actin, p53 and NF-κB were purchased from Santa Rosa (Santa Rosa, CA, USA). Antibodies against p21 and p27 were obtained from Lab Vision (Thermo Fisher Scientific, Fermont, CA, USA). Antibodies against p44/42, phospho p44/42, Bid and Bax were purchased from Cell Signalling Technology (Danvers, MA, USA).

### RNA analysis and PCR

Total RNA was extracted using Tri Reagent (Sigma) according to the manufacturer's protocol. Gene expression was then examined by real-time quantitative RT-PCR, using the GeneAmp RNA PCR kit (Perkin-Elmer, Foster City, CA, USA) and platinum Taq polymerase (Invitrogen, Carlsbad, CA, USA) according to the manufacturers' instructions. Depending on the abundance of the specific mRNA species, 70 to 250 ng total RNA was used as starting template in the RT reaction mixture. To detect amplicon synthesis in the Smart Cycler real-time PCR thermal cycler (Cepheid, Sunnyvale, CA, USA), 0.25 × Cyber green dye (Roche, Indianapolis, IN, USA) was added to the reaction mixture. For quantification, the cycle threshold number (Ct) exhibiting the maximum curve growth rate was determined. The relative gene expression of each sample, normalized to that of glyceraldehyde-3-phosphate dehydrogenase (GAPDH), was calculated by the formula:

### Apoptosis assays

Annexin staining was conducted with the use of a kit (Annexin V-FITC Apoptosis detection kit; BD Pharmingen, San Jose, CA, USA). A sample of 10^6 ^cells per well was split and left to recover for 24 hours before exposure to the proteasome inhibitor. The cells were then treated with indicated concentrations of Bortezomib and BU-32 for 24 hours. After incubation, cells were washed with Dulbecco's phosphate buffered saline (DPBS) (Sigma, St. Louis, MO, USA) and resuspended in 100 μl binding buffer (supplied by the vendor). Cells were stained with annexin V-FITC and propydium iodide according to the manufacturer's protocol before analysis by flow cytometry. Results are presented as mean ± standard deviation of three independent experiments.

### *In vivo *breast cancer metastasis to bone

For bone metastasis experiments, we specifically used MDA-MB-231 cells that had been stably transfected with the gene-encoding green fluorescent protein (GFP) to detect tumor cells in live animals using noninvasive fluorescence imaging. MDA-MB-231-GFP cells (10^5 ^in 100 μl PBS) were injected intracardially. Animals were anesthetized with isofluorane and positioned ventral side up. The left cardiac ventricle was punctured through a percutaneous approach using a 27-gauge needle. Based on an average body weight, Bortezomib (0.02 mg/kg and 0.06 mg/kg body weight) and BU-32 (0.02 mg/kg and 0.06 mg/kg body weight) were given twice a week to animals by subcutaneous injection beginning on the day of tumor cell inoculation (day 0), and continuing until the end of the protocol (day 35). All doses of each drug were given by subcutaneous injection in 50 μl PBS with 0.2% dimethylsulfoxide (vehicle). Control mice received a daily treatment with vehicle only.

On day 35 after tumor cell inoculation, radiographs (MX-20 cabinet X-ray system; Faxitron X-ray Corp. (Lincolnshire, IL, USA)) of anesthetized mice were taken. For radiographs of mice, the animals were anesthetized deeply with isofluorane, laid in a prone position against the film and exposed to an X-ray at 35 KVP for 6 seconds. Films were developed using a RPX-OMAT processor (Eastman Kodak Company, Rochester, NY, USA. All radiographs from nude mice were evaluated in a blinded fashion. The number and area of osteolytic bone metastasis were calculated on radiographs using a computerized image analysis system.

Animals analyzed by radiography were also examined by noninvasive, whole-body fluorescence imaging using a fluorescence scanning system (Nikon-SMZ1500; Nikon, Tokyo, Japan) with a fluorescence stereoscope attached to a Cool SNAP CCD camera (Photometrics, Tucson, AZ, USA). Metastatic lesions in bone were identified on scanned images as fluorescent regions. The area of fluorescent spots was measured using the Bio-Rad densitometer image analysis system (Bio-Rad, Hercules, CA, USA). Anesthetized mice were killed by cervical dislocation after radiography and fluorescence imaging on day 35.

### Human breast cancer cell xenograft

In tumorigenesis studies, MDA-MB-231-GFP tumor cells were implanted subcutaneously into the right flank of 4-week-old to 5-week-old Balb/c female nude mice that had been anesthetized by isofluorane inhalation. The subcutaneous tumor inoculation was performed using a 27-gauge needle attached to a 1 ml syringe containing tumor cells at a concentration of 10^7 ^cells/100 μl PBS. After 10 days, tumor bearing mice were randomly grouped (n = 10) and were treated by daily subcutaneous injection with therapeutic agents (Bortezomib and BU-32) in a dose of 0.02 or 0.06 mg/kg body weight or with vehicle (PBS, control) for 10 days. The tumor size was measured every other day using calipers, and the tumor volumes were calculated according to the standard formula:

The extent of tumor burden per animal was expressed in cubic millimeters. Mice were sacrificed after 28 days of treatment.

### *In vivo *toxicity study

*In vivo *toxicity was performed in Balb/c mice (Harlan Industries, Houston, TX, USA). Mice 2 to 4 weeks old were caged for 1 week before the experiment to acclimatize them to the environment. The mice were fed with standard mice chaw (Harlan Industries) and water *ad libitum*. For the experiment, mice were given a dose range of BU-32 and Bortezomib (0.1 to 0.25 mg/kg twice weekly for 2 weeks) intraperitoneal injection. Each group consisted of 10 mice, and control or untreated mice were given only vehicle (0.3% hydroxyl propyl cellulose). The animals were monitored regularly for external signs of toxicity or lethality.

### Statistical analysis

Unless otherwise stated, all experiments were conducted at least three times and results are expressed as the mean ± standard deviation. Significant differences were calculated by Student's *t *test using SPSS statistical software (IBM Company, Chicago, IL), and significance was achieved when *P *< 0.05.

## Results

### BU-32 inhibits tumor cell proliferation

**Figure 1:**
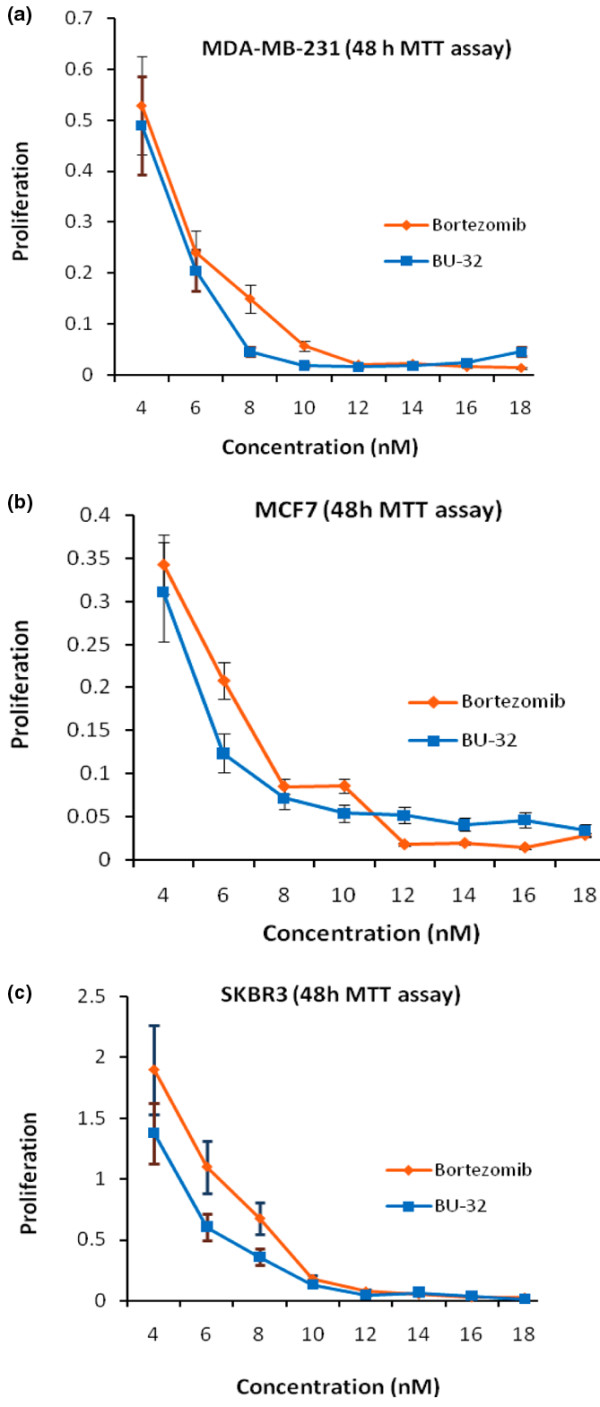
Effect of proteasome inhibitor on proliferation. 3-(4,5-Dimethylthiazol-2-yl)-2,5-diphenyltetrazolium bromide (MTT) assay of **(a) **MDA-MB231, **(b) **MCF7 and **(c) **SKBR3 breast cancer cells treated with different concentrations (4 to 18 nM) of Bortezomib and BU-32 for 48 hours. Results are mean of three independent experiments.

The ubiquitin-proteasome protein degradation pathway plays an essential role in the orderly proteolysis of intracellular proteins. In cancer cells, this pathway affects numerous activities that are important for tumor development. The novel proteasome inhibitor BU-32 shows promising inhibitory activity against MDA-MB-231 cells in the human breast cancer xenograft nude mouse model. We examined the potency of BU-32 at inhibiting the viability of MDA-MB-231 breast cancer cells *in vitro *using the MTT assay. BU-32 reduces the number of viable cells in a dose-dependent manner (Figure 1a) with a half-maximal inhibitory concentration (IC_50_) of 5.8 nM.

Cell viability experiments were also conducted on two other breast cancer cell lines, MCF7 and SKBR3. The results of an MTT assay revealed that BU-32 inhibited cell viability, and proliferation, after 48 hours of treatment in a dose-dependent manner (Figure 1b, c). BU-32 therefore exhibits strong cytotoxicity on MCF7 cells (IC_50 _= 5.8 nM) and on SKBR3 cells (IC_50 _= 5.7 nM). These values are comparable with those obtained with PS-341 for the MDA-MB-231 (IC_50 _= 5.9 nM), MCF7 (IC_50 _= 6.4 nM) and SKBR3 (IC_50 _= 6.8 nM) cell lines.

### BU-32 selectively inhibits proteasome chymotrypsin-like activity

**Figure 2:**
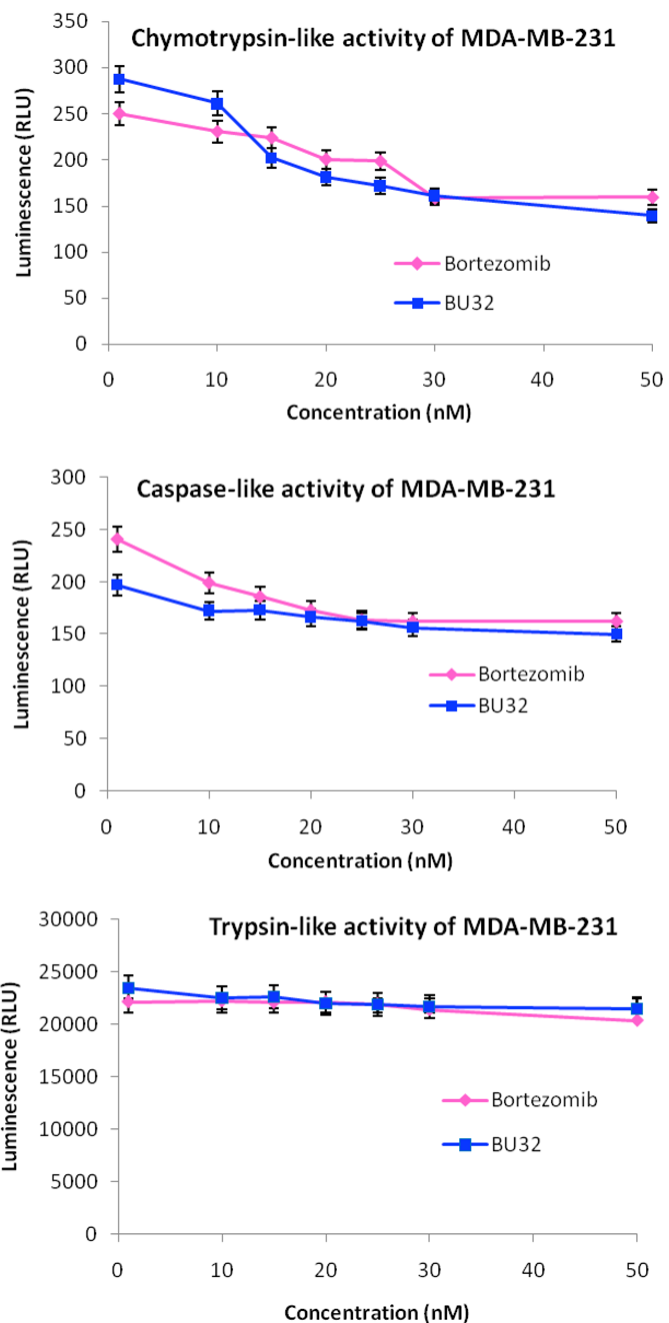
Effect of BU-32 on proteasomal catalytic activity of MDA-MB-231 cells. Proteasome inhibition by Bortezomib and BU-32 in the MDA-MB-231 breast cancer cell line was measured. The cells were treated with different concentrations of proteasome inhibitor from 1 to 50 nM for 24 hours, and were then analyzed for the inhibition of chymotryptic-like, caspase-like, and tryptic-like intracellular proteasome activities. Results are mean of three individual experiments. RLU: Relative Luminescence Units.

**Figure 3:**
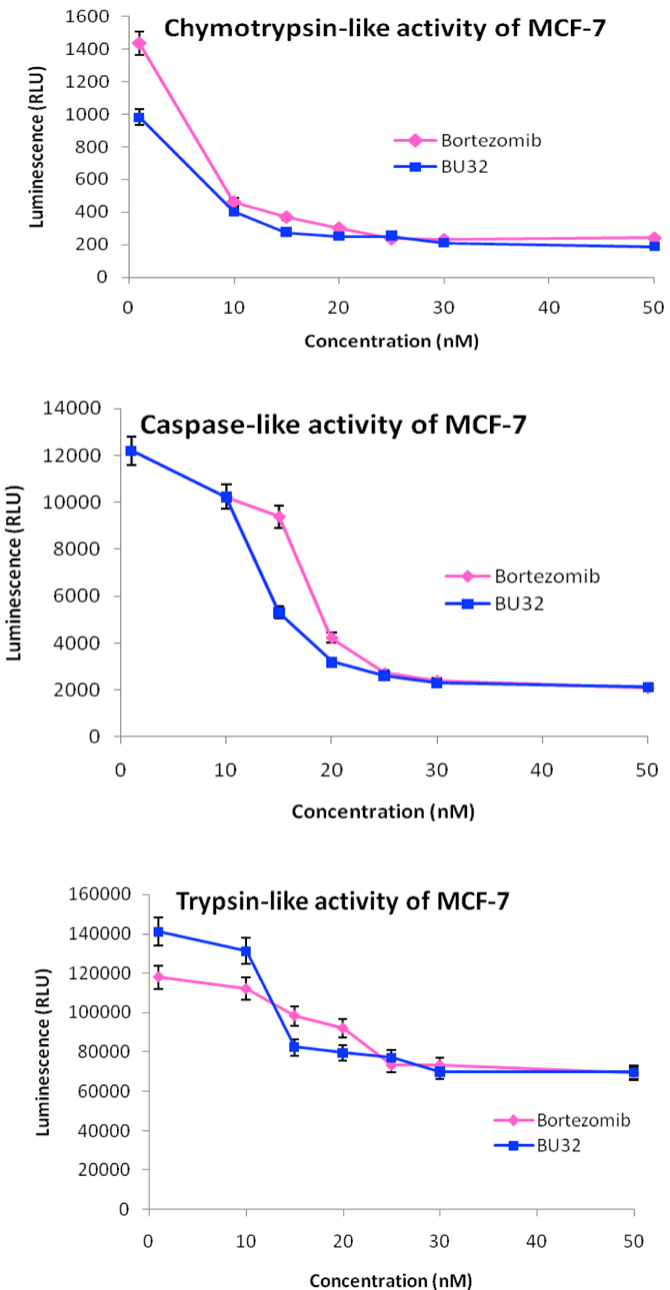
Effect of BU-32 on proteasomal catalytic activity of MCF-7 cells. Proteasome inhibition by Bortezomib and BU-32 in the MCF-7 breast cancer cell line was measured. The cells were treated with different concentrations of proteasome inhibitor from 1 to 50 nM for 24 hours, and were then analyzed for the inhibition of chymotryptic-like, caspase-like, and tryptic-like intracellular proteasome activities. Results are mean of three individual experiments. RLU: Relative Luminescence Units.

**Figure 4:**
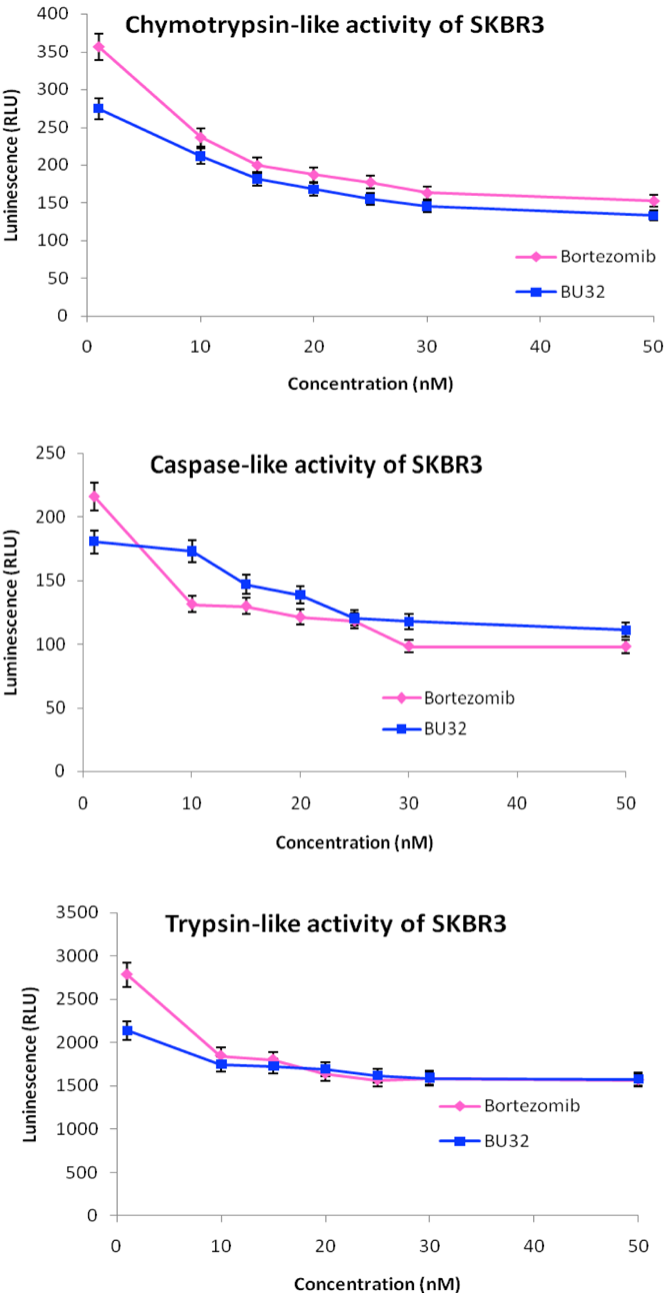
Effect of BU-32 on proteasomal catalytic activity of SKBR3 cells. Proteasome inhibition by Bortezomib and BU-32 in the SKBR3 breast cancer cell line was measured. The cells were treated with different concentrations of proteasome inhibitor from 1 to 50 nM for 24 hours, and were then analyzed for the inhibition of chymotryptic-like, caspase-like, and tryptic-like intracellular proteasome activities. Results are mean of three individual experiments. RLU: Relative Luminescence Units.

The proteasome plays a central role in regulation of cell cycle, proliferation, cell death, angiogenesis, metastasis and resistance to chemotherapy and radiation therapy. Bortezomib is a potent and selective inhibitor of the chymotrypsin-like activity of the 20S proteasome. To evaluate the potency and selectivity of BU-32 for the three proteasome catalytic active sites, we monitored the rates of fluorogenic peptide substrate hydrolysis by the proteasome in intact cells. Our results showed that BU-32 is potent and highly selective for the inhibition of the chymotrypsin-like and caspase-like activities of the proteasome (Figures 2 to 4). Incubation of a panel of breast cancer cell lines (MDA-MB-231, MCF7, and SKBR3) with BU-32 for 24 hours resulted in a dose-dependent inhibition of all three proteasome catalytic activities, with the chymotrypsin-like activity exhibiting the greatest sensitivity for MDA-MB-231 cells (Figure 2), MCF7 cells (Figure 3), and SKBR3 cells (Figure 4).

### BU-32 upregulates apoptosis and downregulates NF-κB expression

**Figure 5:**
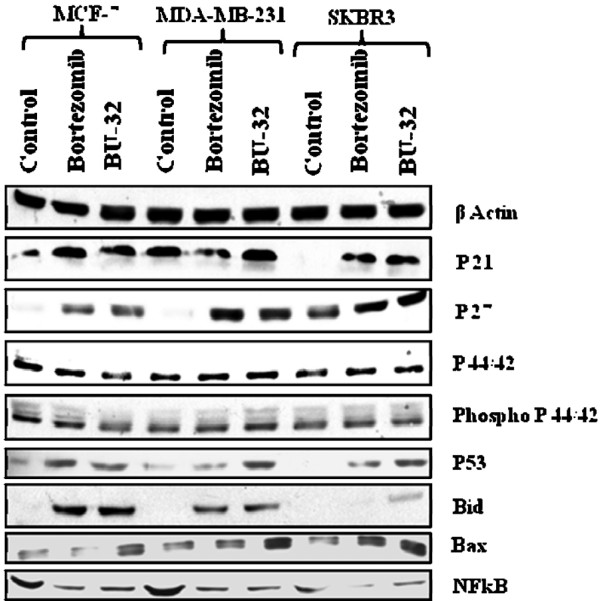
BU-32 upregulates apoptosis and downregulates NF-κB expression. BU-32 exposure induces accumulation of proapoptotic markers, cell-cycle-dependent kinase inhibitors and the p53 tumor suppressor gene, and downregulates NF-κB expression in breast cancer cells. Western blot analysis of total cell extracts of all three breast cancer cell lines shows the effect of treatment with Bortezomib and BU-32 on the expression level of the cyclin-dependent kinase inhibitor proteins p21 and p27, tumor suppressor p53, proapoptotic genes Bid and Bax, anti-apoptotic NF-κB, and cell cycle regulatory protein p44/42, phospho p44/42. Breast cancer cell lines were examined after 24 hours of exposure to the proteasome inhibitors (5 nM) and a series of western blots using specific antibodies was performed to detect relative levels of proteins. Results are the average of three independent measurements.

Exposure to BU-32 induces the upregulation of proapoptotic markers, cell-cycle-dependent kinase inhibitors and the tumor suppressor gene, and downregulates NF-κB expression in tumor cell lines. The proteasome is a hub for the regulation of many cellular signaling pathways, and proteasome inhibition therefore appears to induce apoptosis through a number of mechanisms. Inhibition of the proteasomal chymotrypsin-like activity, but not trypsin-like activity, has been shown to be associated with apoptosis induction in cancer cells. To further investigate the pathways activated by BU-32, western blot analysis was carried out on cell lysates prepared from MDA-MB-231, MCF7 and SKBR3 cell lines after 24 hours of treatment with therapeutic compound. Exposure to BU-32 upregulated apoptotic markers including Bid and Bax and accumulation of cell cycle inhibitor proteins p21 and p27 as well as stabilization of tumor suppressor protein p53 (Figure 5). The effect of BU-32 on accumulation of these markers was comparable with that observed with Bortezomib, and is consistent with the observed effects of BU-32 on cell viability and apoptosis.

The expression of NF-κB is downregulated by BU-32. NF-κB has been shown to inhibit apoptosis by promoting the expression of anti-apoptotic proteins such as Bcl-2 and Bcl-xL. Inhibition of NF-κB by BU-32 can therefore lead to activation of proapoptotic proteins that are normally bound and inhibited by Bcl-2 and Bcl-xL, such as Bax and Bid. The disruption of cell cycle and apoptosis regulators can alter cellular response to proteasome inhibitor.

### BU-32 downregulates angiogenic marker genes

Exposure to BU-32 causes downregulation of a wide array of angiogenic marker genes in breast cancer cell lines. Angiogenesis is essential for the development and progression of both hematologic and solid malignancies, where new vessels deliver oxygen and nutrients to the burgeoning tumor. As such, agents that block angiogenesis are highly desired in cancer therapy. Suppression of angiogenesis is an important component of the anti-cancer activity of Bortezomib, and is thought to largely occur via inhibition of NF-κB - which induces the expression of many proangiogenic factors, including vascular endothelial growth factor (VEGF).

**Figure 6:**
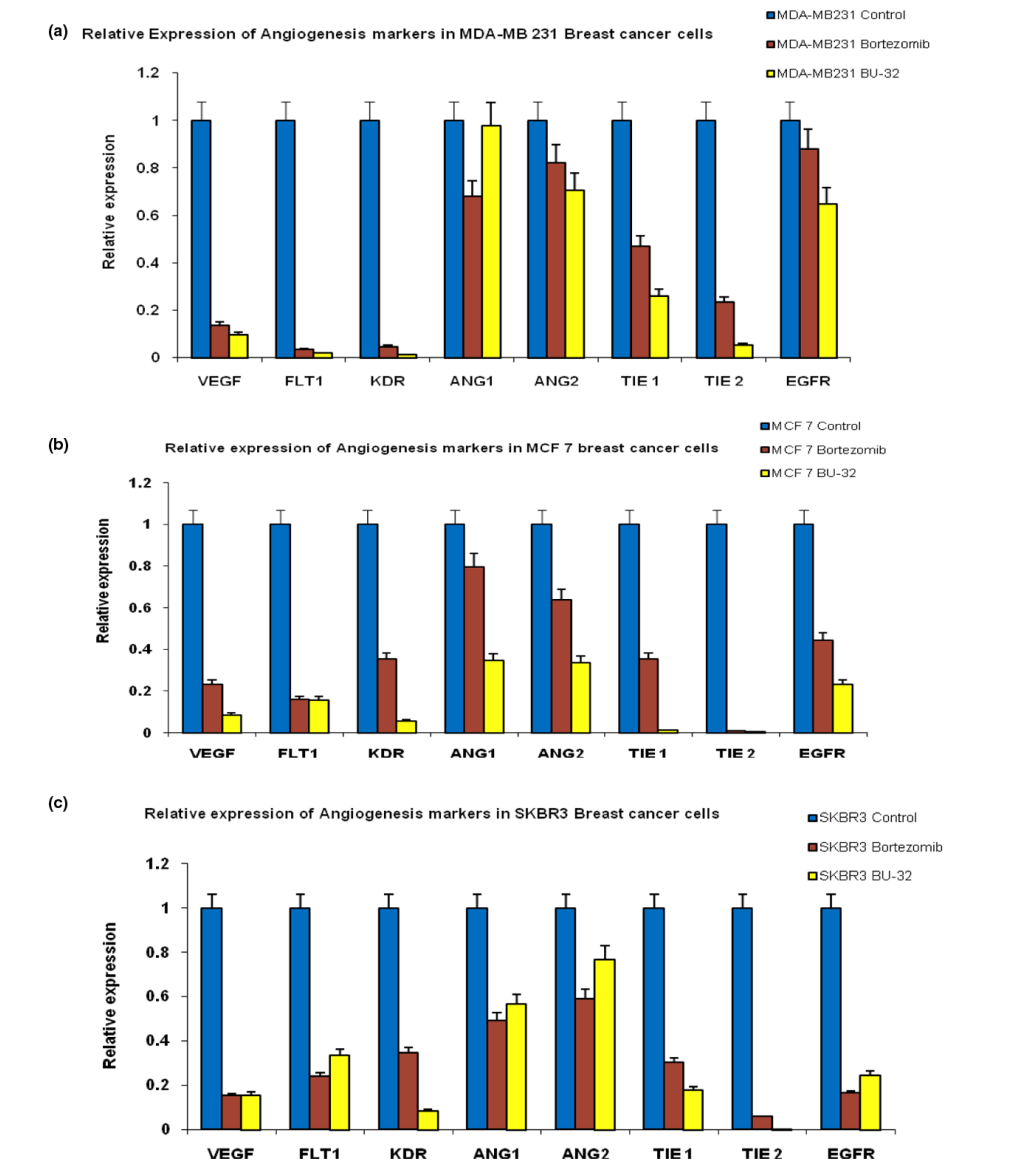
BU-32 downregulates the expression of genes involved in angiogenesis. Expression levels of vascular endothelial growth factor (VEGF), VEGF-receptor 1 (FLT1), kinase-insert domain-containing receptor (KDR), Ang1, Ang2, Tie1, Tie2, and epidermal growth factor receptor (EGFR) genes were measured by RT-PCR in **(a) **MDA-MB-231, **(b) **MCF7 and **(c) **SKBR3 breast cancer cell lines exposed to proteasome inhibitors BU-32 and Bortezomib, as evaluated by real-time RT-PCR. The RT-PCR was performed on 200 ng total RNA extracted with Trizol reagent from breast cancer cell lines and was cultured for 24 hours with 5 nM concentrations of Bortezomib and BU-32. Results are mean ± standard deviation of three independent determinations.

We investigated whether exposure to BU-32 affects the expression of genes involved in the angiogenic cascade, such as VEGF, VEGF-receptor 1 (FLT1), the angiopoietin-Tie system (Ang1, Ang2, Tie1, and Tie2), epidermal growth factor receptor, and kinase insert domain receptor (KDR) in the MDA-MB-231 (Figure 6a), MCF-7 (Figure 6b), and SKBR3 (Figure 6c) cell lines, and compared the effect with that of Bortezomib. We showed that exposure to BU-32 induces a significant downregulation of these angiogenic markers in all three cell lines and that the effect was generally more pronounced for BU-32 than for Bortezomib. Breast cancer cell lines treated with BU-32 also displayed a marked decrease in a wide array of angiogenic marker genes (Figure 6). Proteasome inhibitors block activation of NF-κB, thereby inhibiting the secretion of VEGF which is associated with angiogenesis.

### Exposure to BU-32 induces apoptosis in tumor cell lines

**Figure 7:**
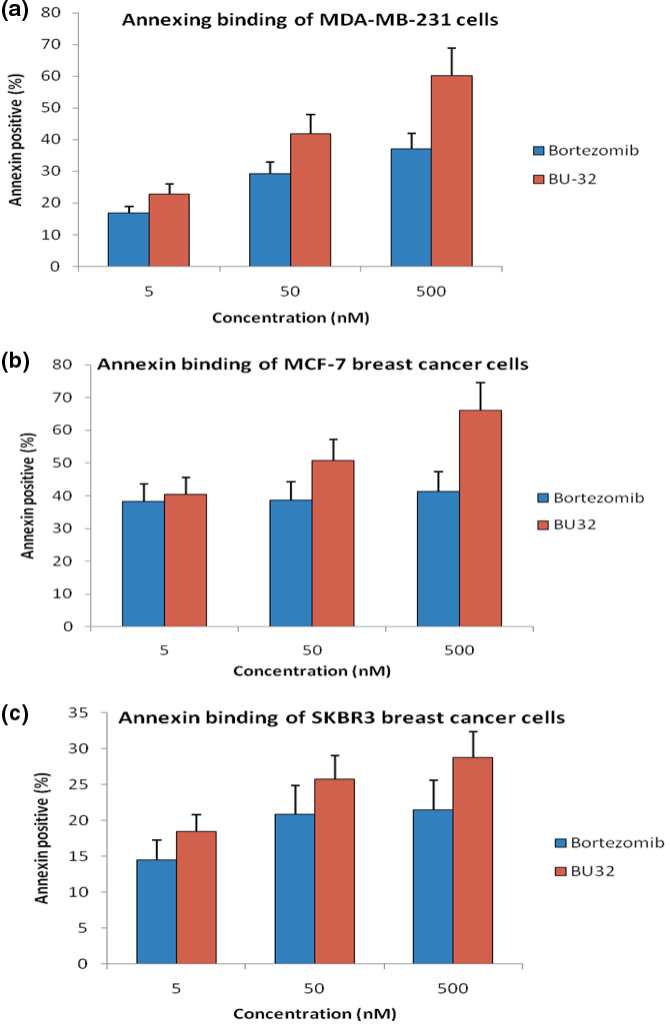
Exposure to BU-32 induces apoptosis in tumor cell lines. Annexin staining was conducted with the use of a kit (Annexin V--FITC Apoptosis detection kit; BD Pharmingen (San Jose, CA, USA)). The breast cancer cells were treated with 5 nM, 50 nM, and 500 nM Bortezomib and BU-32 for 24 hours. Cells were stained with annexin V--FITC and propydium iodide according to the manufacturer's protocol before analysis by flow cytometry. Results are mean ± standard deviation of three independent experiments for **(a) **MDA-MB-231, **(b) **MCF7 and **(c) **SKBR3 cell lines.

Annexin V-FITC was used to quantitatively determine the percentage of cells within a population that were actively undergoing apoptosis. Our results indicate that both Bortezomib and BU-32 proteasome inhibitors induce apoptosis in different breast cancer cells (MDA-MB-231, MCF7 and SKBR3). The effect is more prominent in the case of BU-32-treated cells and the percentage of apoptotic cells increases in a dose-dependent manner (Figure 7).

### BU-32 treatment is efficacious in blocking breast tumor growth and bone metastases

**Figure 8:**
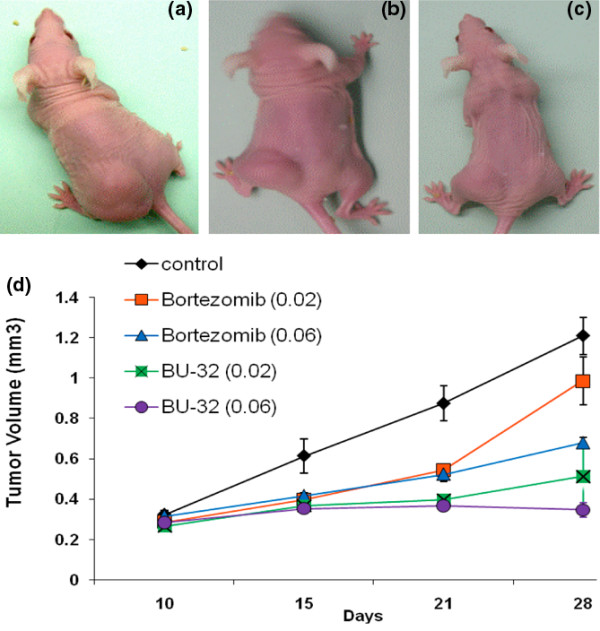
Effect of proteasome inhibitor on established human breast cancer cell (MDA-MB-231-GFP) tumor xenograft. Subcutaneous tumors were generated by injecting 1 × 10^7 ^MDA-MB-231-GFP cells into the flanks of nude mice. Tumors were established for 10 days before the initiation of therapy. Representative images were taken under microscope at the end of week 3 of therapy and show results for **(a) **vehicle-treated controls, **(b) **Bortezomib-treated mice and **(c) **BU-32-treated mice. Doses of 0.02 and 0.06 mg/kg body weight were used for Bortezomib and BU-32. **(d) **Tumor volumes were measured 1, 2, and 3 weeks after initiation of therapy. Results are mean ± standard deviation (n = 10).

We evaluated the effect of BU-32 in *in vivo *xenograft tumor studies using MDA-MB-231-GFP cells, and found that there was localized accumulation of breast cancer cells in the tumors of control mice but the tumor burden was significantly reduced in the group treated with BU-32 (0.06 mg/kg). The tumor volume was reduced from 1.2 cm^3 ^(control) to 0.3 cm^3 ^(BU-32 0.06 mg/kg) on day 28 of the experiment (Figure 8a to 8c). Both Bortezomib and BU-32 decreased the tumor burden in a dose-dependent manner (Figure 8d).

**Figure 9:**
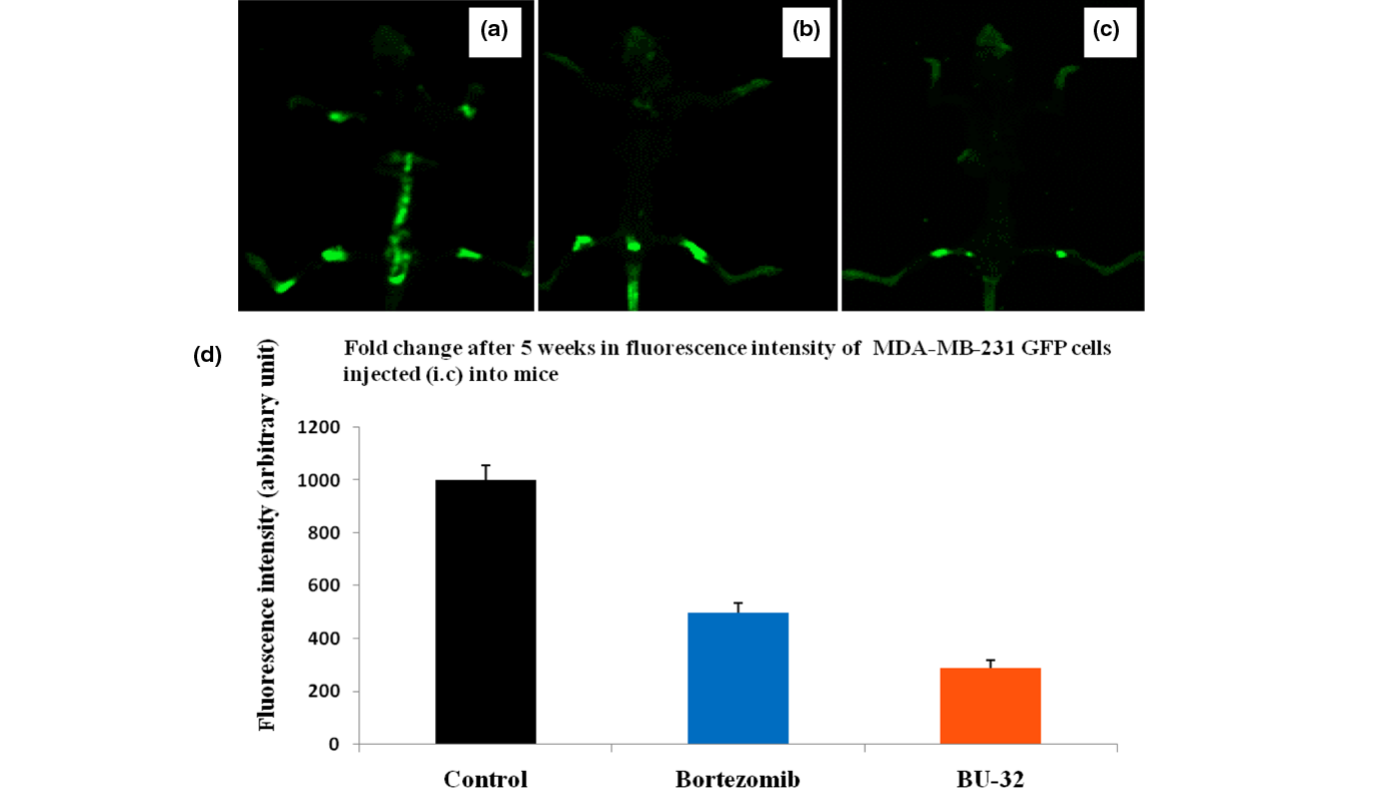
Inhibition of breast cancer metastasis in bone by proteasome inhibitor. Mice were injected intracardially with 1 × 10^5 ^MDA-MB-231-GFP cells. The mice were then treated with Bortezomib and BU-32 using a dose of 0.06 mg/kg body weight or with vehicle PBS as control. Whole-body high-resolution fluorescence images of MDA-MB231-GFP-expressing breast cancer metastatic cells in the skeleton of live intact animals were measured after 5 weeks. Representative fluorescence images are shown for **(a) **the control group, **(b) **Bortezomib-treated mice and **(c) **BU-32-treated mice. **(d) **The fluorescence intensities were quantified. GFP, green fluorescent protein; i.c., intracisternal.

The anti-tumor activity of BU-32 was evaluated and compared with Bortezomib in Balb/c nude mice bearing established human tumor xenografts derived from the tumor cell line MDA-MB-231-GFP. The dosing (0.06 mg/kg) regimen was therefore chosen to compare the effects of BU-32 and Bortezomib on the formation of MDA-MB-231-GFP breast cancer bone metastases. Noninvasive fluorescence imaging on day 35 after tumor cell injection showed that metastatic mice treated with Bortezomib and BU-32 had statistically significantly lower metastatic lesions compared with control mice (Figure 9a to 9c). Radiographic analysis on day 35 after intracardial tumor cell injection revealed that metastatic animals treated with BU-32 had 75% lower metastatic tumor burden than those of tumor-bearing mice treated with the vehicle (Figure 9d).

### *In vivo *toxicity study

*In vivo *toxicity studies were conducted in Balb/c mice at two different doses of BU-32 and Bortezomib (0.1 to 0.25 mg/kg twice weekly for 2 weeks). Each group consisted of 10 mice and the control or untreated group were given only vehicle (0.3% hydroxylpropyl cellulose). The animals were monitored regularly for external signs of toxicity or lethality. The group of mice administered 0.25 mg/kg Bortezomib showed one death on day 3 and a total of nine deaths by day 9 (Table 1). On the other hand, the same dose (0.25 mg/kg) of BU-32 showed only two deaths but the rest of the mice appeared tired and showed increased furring as in the remaining Bortezomib group. The furring and weakness or fatigue was sustained in mice treated with the lower dose of Bortezomib (0.1 mg/kg) and two deaths were noticed. Interestingly, the BU-32 group treated with 0.1 mg/kg drug showed no lethality or any other external signs of toxicity.

**Table 1:** *In vivo *toxicity study of BU-32 compared with Bortezomib

	Untreated control	BU-32		Bortezomib	
		
		0.1 mg/kg	0.25 mg/kg	0.1 mg/kg	0.25 mg/kg
Total number of mice/group	10	10	10	10	10
Week 1	10	10	10	10	9
Week 2	10	10	8	8	1

Ten mice in each group were treated twice a week for 2 weeks with vehicle (control) or with 0.1 and 0.25 mg/kg BU-32 and Bortezomib. BU-32-treated mice showed more tolerance than Bortezomib at higher dose (0.25 mg/kg), and the lower dose of BU-32 (0.1 mg/kg) was completely safe to the animals after 2 weeks of treatment.

## Discussion

In the present article we show that the novel diboronated analog of Bortezomib BU-32 (NSC D750499-S) is a potent and selective inhibitor of the chymotrypsin-like activity of the 20S proteasome and has potent *in vitro *anti-tumor activity against a panel of breast cancer cells as well as *in vivo *efficacy in mouse xenograft and metastasis models. In the present study, we first compared the cytotoxicity of BU-32 with Bortezomib, which has been validated clinically for the treatment of multiple myeloma and non-Hodgkin's lymphoma, and works by reversibly inhibiting the effects of the proteasome and by inducing apoptosis in several tumor cell lines and animal models [13-15]. The results of our MTT experiments demonstrate that BU-32 induces strong growth arrest at clinically relevant concentrations *in vitro *( < 10 nM) with MDA-MB-231, MCF-7, and SKBR-3 cell lines, and that MDA-MB-231-GFP tumor xenografts treated with intravenous BU-32 displays substantial inhibition of proliferation *in vivo *in a dose-dependent manner. Interestingly, the cytotoxicity of BU-32 against the panel of three breast cancer cell lines is comparable with that of Bortezomib in spite of the more bulky size of BU-32 and the possibility of steric hindrance at the binding pocket of the proteasome. While a definitive evidence for the nature of the interactions of BU-32 with the binding pockets of the proteasome awaits further studies, it is clear from our *in vitro *and *in vivo *data that there is reasonably good interaction with the active site given the observed potency of BU-32 in these cell lines.

We tested the active site selectivity of BU-32 in our panel of breast cancer cell lines, and found that it blocks all three proteasomal activities in the MDA-MB-231, MCF-7, and SKBR3 cell lines - with the chymotrypsin-like and caspase-like activities being the most predominant, and to a lesser degree the trypsin-like activity. Our observation is consistent with the active site selectivity profile reported for Bortezomib [3,17,33-38].

Proteasome-induced cytotoxicity could potentially result from multiple events, including the stabilization and deregulated function of cyclins, cell-cycle-dependent kinase inhibitors, tumor suppressor proteins, IκB, proapoptotic proteins, and a large number of other proteins associated with cell cycle progression[14]. Indeed, proteasome inhibition has been shown to stabilize the cyclin-dependent kinase inhibitors p21 and p27, the tumor suppressor p53, and the proapoptotic proteins Bid and Bax [15,39-45]. The increased levels of activated p21, p27, p53, Bid, and Bax result in inhibition of cell cycle progression and/or promotion of apoptosis in response to Bortezomib [45]. As with the proteasome inhibitor Bortezomib, BU-32 exposure leads to accumulation of the cell-cycle-dependent kinase inhibitors p21 and p27, the p53 tumor suppressor gene and the proapoptotic proteins Bid and Bax, and downregulation of NFκB at the protein level in MCF-7, MDA-MB-231, and SKBR3 cell lines. There is no significant difference in the levels of expression of the mitogen-activated protein kinases p42, p44, phospho-p42 and phospho-p44 compared with the controls. Although the p21 and p27 levels increased in all three cell lines for both BU-32 and Bortezomib, our results show that the effect is cell line dependent.

These observations are consistent with an earlier report that the effect of proteasome inhibition on different markers varies by drug, cell line, and time point of analysis [46]. BU-32 exposure caused greater accumulation of Bax in all three cell lines. It is known that p53 plays important roles in cell cycle regulation and apoptosis [47-49]. Exposure to BU-32 resulted in accumulation of the p53 tumor suppressor gene in all three breast cancer cell lines we studied. The effect was more pronounced for BU-32 in the cell lines MDA-MB-231 and SKBR3 than in MCF-7 cells compared with Bortezomib. Proteasome inhibitor-induced apoptosis has been described as p53 dependent [12]. Other reports have shown that sensitivity to proteasome inhibition was partially dependent on the p53 status of the breast [50] and lung cancers *in vitro *[47], but Bortezomib-induced apoptosis was p53 independent in prostate cancer cells [14], multiple myeloma [15], and colon cancer cells [51]. As with Bortezomib [45], the degree of variability in the sensitivity to BU-32 with respect to p53 status appears to be cell line dependent.

We next examined whether exposure to BU-32 downregulated the transcription factor NF-κB. We found that BU-32 downregulated the expression of NF-κB in our panel of breast cancer cell lines and that the effect was comparable with Bortezomib. The initial rationale for the use of Bortezomib in multiple myeloma was inhibition of NF-κB activity by blocking proteasomal degradation of inhibitor of κBα [52]. Interestingly, recent reports indicate that there is a paradigm shift in myeloma with respect to ascribing the mechanism of Bortezomib's anti-tumor activity to NF-κB inhibition [53], and suggest that Bortezomib-induced cytotoxicity cannot be fully attributed to inhibition of canonical NF-κB activity [54].

We also investigated whether exposure to BU-32 affects the expression of genes involved in the angiogenic cascade, such as VEGF, VEGF-receptor 1 (FLT1), the angiopoietin-Tie system (Ang1, Ang2, Tie1, and Tie2), epidermal growth factor receptor, and KDR in MCF7, MDA-MB-231, and SKBR3 cell lines, and compared the effect with that of Bortezomib. Their expression in endothelial cells increases with the angiogenic switch and persists throughout angiogenesis - except during vessel stabilization, when inhibitors prevail [55]. It has been shown that VEGF triggers blood vessel formation [56]. Ang1 and Ang2 are mandatory for vessel sprouting and remodeling [57]. Ang1 promotes sprouting in the presence of VEGF, induces branching networks with the typical organization of mature vessels, and stabilizes perivascular endothelial cell interactions [56]. Ang2 exerts a vessel destabilizing effect, which allows VEGF-mediated vascular reorganization [56]. The angiopoietin-Tie2 system appears to govern maturation and stabilization of blood vessels. It is well understood from previous studies that VEGF mainly binds to and activates two different receptor tyrosine kinases: fms-like tyrosine kinase 1 (Flt-1) and KDR [58]. It is also reported in human vascular endothelial cells that KDR mediates the majority of VEGF transcription-regulating activities [59] and recent studies have demonstrated that KDR mediates the mitogenic, chemotactic, tubulogenic, and survival activities of VEGF [60]. In the present article we showed that exposure to BU-32 induces a significant downregulation of these angiogenic markers in all three cell lines and that the effect was generally more pronounced for BU-32 than Bortezomib. These data suggest that BU-32 exerts anti-angiogenic activity through the downregulation of genes required for growth of endothelial cells, and that angiogenesis is a possible mechanism of its cytotoxicity.

We also investigated the anti-breast cancer activity of BU-32 *in vivo *in a human breast cancer mouse model. In a xenograft model of breast cancer, BU-32 treatment resulted in significant inhibition of tumor growth. We also showed that BU-32 significantly reduces breast-cancer-initiated bone metastasis *in vivo*. These effects were not associated with any significant toxicity when compared with Bortezomib. We also showed from our toxicity study that BU-32 has a broader safe dosing range compared with Bortezomib. From this toxicity study, BU-32 showed a promising tolerance profile and therefore can offer a wide range of therapeutic index. Even though BU-32 appears to be an appealing therapeutic candidate, further pharmacokinetic studies as well as pharmacodynamic studies need to be conducted to validate the activity and safety profiles.

## Conclusions

Taken together, the results described in the present study demonstrate that the novel proteasome inhibitor BU-32 possesses several *in vitro *and *in vivo *properties that suggest it may be a promising anti-cancer therapeutic, and provide the rationale for further clinical trials of BU-32 either alone or in combination with other therapies in breast cancer patients. BU-32 is a highly selective and potent inhibitor of 26S proteasome. Preclinical studies currently being conducted by the National Cancer Institute against a panel of 60 cell lines show that BU-32 has broad anti-tumor activity (data not shown), and numerous biochemical studies are currently ongoing to investigate its efficacy as a single agent and in combination with other active anti-tumor agents against a variety of malignancies. Our preclinical observation that treatment with BU-32 may be associated with less toxicity suggests its promise as an anti-cancer agent.

## Abbreviations

Ct: cycle threshold number; DMEM: Dulbecco's modified Eagle's medium; FBS: fetal bovine serum; FITC: fluorescein isothiocyanate; GAPDH: glyceraldehyde-3-phosphate dehydrogenase; GFP: green fluorescent protein; IC_50_: half-maximal inhibitory concentration; KDR: kinase-insert domain-containing receptor; MTT: 3-(4,5-dimethylthiazol-2-yl)-2,5-diphenyltetrazolium bromide; NF: nuclear factor; PBS: phosphate-buffered saline; PCR: polymerase chain reaction; RT: reverse transcriptase; VEGF: vascular endothelial growth factor.

## Competing interests

The authors declare that they have no competing interests.

## Authors' contributions

JKA conceived, designed and coordinated the study, provided study material and helped to write the manuscript. HBN performed the *in vitro *and *in vivo *studies. SSR assisted in writing the manuscript. BS synthesized BU-32. RRT provided experimental support and proofread the manuscript. All authors read and approved the final manuscript.
